# Relapse of Acute Myeloid Leukemia after Allogeneic Stem Cell Transplantation: Prevention, Detection, and Treatment

**DOI:** 10.3390/ijms20010228

**Published:** 2019-01-08

**Authors:** Christina Rautenberg, Ulrich Germing, Rainer Haas, Guido Kobbe, Thomas Schroeder

**Affiliations:** Department of Hematology, Oncology and Clinical Immunology, University of Duesseldorf, Medical Faculty, 40225 Duesseldorf, Germany; Christina.Rautenberg@med.uni-duesseldorf.de (C.R.); Germing@med.uni-duesseldorf.de (U.G.); Haas@med.uni-duesseldorf.de (R.H.); Kobbe@med.uni-duesseldorf.de (G.K.)

**Keywords:** acute myeloid leukemia, allogeneic transplantation, relapse, maintenance, minimal residual disease, salvage therapy

## Abstract

Acute myeloid leukemia (AML) is a phenotypically and prognostically heterogeneous hematopoietic stem cell disease that may be cured in eligible patients with intensive chemotherapy and/or allogeneic stem cell transplantation (allo-SCT). Tremendous advances in sequencing technologies have revealed a large amount of molecular information which has markedly improved our understanding of the underlying pathophysiology and enables a better classification and risk estimation. Furthermore, with the approval of the FMS-like tyrosine kinase 3 (FLT3) inhibitor Midostaurin a first targeted therapy has been introduced into the first-line therapy of younger patients with FLT3-mutated AML and several other small molecules targeting molecular alterations such as isocitrate dehydrogenase (IDH) mutations or the anti-apoptotic b-cell lymphoma 2 (BCL-2) protein are currently under investigation. Despite these advances, many patients will have to undergo allo-SCT during the course of disease and depending on disease and risk status up to half of them will finally relapse after transplant. Here we review the current knowledge about the molecular landscape of AML and how this can be employed to prevent, detect and treat relapse of AML after allo-SCT.

## 1. Introduction

Allogeneic stem cell transplantation (allo-SCT) is besides the use of conventional chemotherapy the second backbone of therapy for patients with acute myeloid leukemia (AML) who are eligible for intensive therapy. Allo-SCT offers the highest potential for long-term survival as postremission therapy in those with an intermediate or high-risk disease and as salvage therapy in those with relapsed or resistant disease, regardless of prognostic biological characteristics [1]. Still, despite substantial improvements regarding the reduction of non-relapse mortality during the last decades [2] relapse represents the major cause of treatment failure and up 50% of AML patients finally relapse after allo-SCT depending on disease status and characteristics [3]. Their prognosis is generally dismal, since many of them, in particular those with early relapse, can either not tolerate or are refractory to low-dose or intensive chemotherapy that are usually applied in this situation. Furthermore, even with cellular therapies such as donor lymphocyte infusions [4] and second transplantation in selected cases or the use of investigational agents only a minority of patients can be rescued in the long run. As a consequence, 2-year survival rates of patients with AML who relapsed after allo-HCT are below 20% independent from the choice of salvage therapy [5,6,7,8]. This poor prognosis and the limited success of salvage therapies imply the need for novel strategies to prevent, detect and to treat relapse.

**Figure 1:**
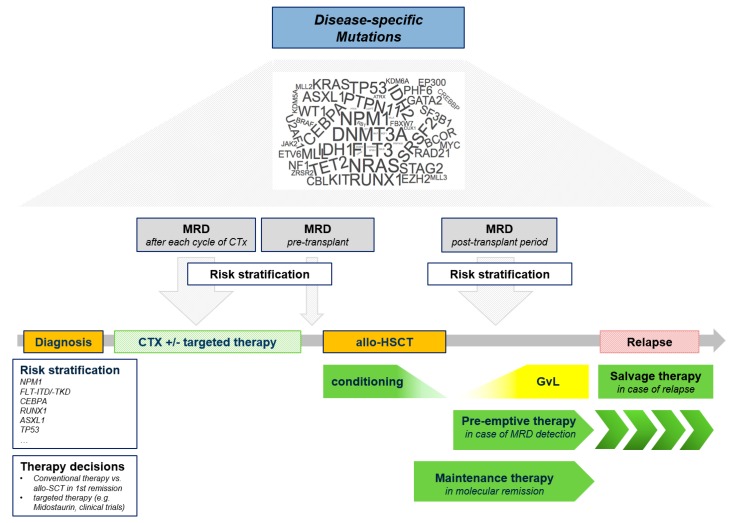
Clinical use of molecular information to prevent, detect, and treat relapse after allogeneic stem cell transplantation (allo-SCT). MRD (minimal or measurable residual disease); NPM1 (Nucleophosmin); FLT3-ITD (FMS-like tyrosine kinase 3-internal tandem duplication); FLT3-TKD (FMS-like tyrosine kinase 3-tyrosine kinase domain); CEBPA (CCAT/enhaner-binding protein alpha); RUNX1 (Runt-related transcription factor 1); ASXL1 (additional sex comb-like 1); TP53 (Tumor Protein 53); allo-SCT (allogeneic stem cell transplantation); GvL (Graft-versus-Leukemia); CTx (Chemotherapy).

During the last two decades extensive advances in molecular techniques, in particular massive parallel or next generation sequencing, have facilitated the discovery of a large number of molecular aberrations in patients with AML. These enabled a comprehensive view on the molecular landscape and have substantially moved forward our understanding of the pathophysiology of AML [9]. Furthermore, this knowledge now allows us to group patients into distinct molecular subgroups, to perform risk estimation and to adjust our post-remission treatment strategy, e.g., high-dose AraC-based consolidation or allo-SCT [1,10,11]. In addition, we are using more and more molecular information identified at the time of diagnosis to track the disease at the lowest level as technically possible (=minimal or measurable disease, MRD) during and after therapy by molecular techniques in order to adapt therapy if needed [12,13,14,15] (Figure 1). Finally, new therapies targeting in particular molecular aberrations (e.g., FMS-like tyrosine kinase 3 (FLT3) and isocitrate dehydrogenase (IDH) inhibitors) [16,17,18], anti-apoptotic pathways (e.g., b-cell lymphoma 2 (BCL) inhibitors) [19,20,21], but also surface antigens on leukemic cells (e.g., gemtuzumab ozogamicin) [22,23] and new pharmacokinetic compositions of classical cytostatic drugs (e.g., CPX-351) [24] have entered the therapeutic arena. Here, we aim to give an overview how this knowledge about the molecular landscape can be integrated into the care of patients with AML in order to prevent, detect and treat relapse after allo-SCT. Giving the rapidly evolving evidence in this field we are aware that we may have not been able to cover all relevant work. Indeed, we have selected relevant articles mainly based on the intention of current applicability or availability in the near future.

## 2. Mutational Landscape and Pathophysiology of AML

Large, comprehensive genomic discovery studies and advances in stem cell biology have tremendously improved our understanding of the pathogenesis and the molecular heterogeneity of AML. Even though not completely understood, AML is believed to originate from a hematopoietic stem or progenitor cell (HSPC) that acquires genomic and molecular alterations. As a consequence of this, HPSC gains stem-cell like properties of unrestricted self-renewal thereby becoming capable to maintain the malignant clone [25,26]. It seems to be the case that specific mutations involved in epigenetic regulation, such as mutations in DNA-methyltransferase 3a (DNMT3A), Tet methylcytosine dioxygenase 2 (TET2) and Additional sex comb-like 1 (ASXL1) genes, occur early during leukemogenesis as founder events prior to leukemogenic alterations (such as NPM1 or other mutations in signaling molecules) [27,28,29]. Such early mutations in pre-leukemic HSPC may even be present in patients decades before leukemia evolves or can be found in approximately 10% of healthy individuals older than 65 years without evidence for a hematological malignancy. The latter state has been termed “clonal hematopoiesis of indeterminate potential” (CHIP) acknowledging the aspect that those patients have, besides an additional association with atherosclerotic cardiovascular disease, an elevated risk to develop a myeloid or other hematological malignancy. Notably, the risk to develop overt hematological malignancy from CHIP state is estimated to be between 0.5 to 1.0% per year and thereby comparable with other pre-malignant states such as progression from monoclonal gammopathy of undetermined significance (MGUS) to multiple myeloma [30,31].

Overall, the number of mutations per AML genome seems to be lower than for most other cancers with an average of 13 coding mutations (single nucleotide variants and insertion/deletions) per person. These recurrent mutations occur in commonly deregulated pathways such as DNA methylation associated genes, spliceosome-complex genes, cohesion-complex genes, chromatin-modifying genes, signaling genes and 96% patients of the AML patients have at least one driver mutation in one of these genes [9,29,32]. This genomic and combinatorial diversity represents the background that drives the heterogeneity in phenotype and prognosis of patients with AML. Together with the results from conventional cytogenetics the information about these mutations is the essential component to diagnose AML according to the WHO 2016 classification and to estimate the prognosis according to the ELN Genetic Risk stratification [32,33].

To further refine the classification and prognostication Papaemannuil and colleagues recently performed a comprehensive sequencing analysis of 111 genes in 1540 patients. Hereby, they were able to define in addition to 8 established AML subsets three new subtypes which included AML with spliceosome mutations, AML with Tumor Protein 53 (TP53) mutations and AML with IDH2^R172^ mutations [32].

Besides a better disease classification, risk stratification and tailoring of therapies with regard to the decision between consolidation with conventional chemotherapy and allo-SCT this knowledge about the underlying molecular biology enables high-resolution tracking of the disease after transplant by quantitative assessment of molecular markers and targeted therapeutic interventions.

## 3. Methods for Relapse Detection—Monitoring of Minimal Residual Disease (MRD)

While AML patients, who relapse after transplantation, generally have a dismal prognosis, recent studies clearly showed that treatment is more effective if started at molecular relapse with low disease burden rather than at hematological relapse [8,34,35]. Thus, successful therapeutic intervention requires an early detection of imminent relapse ideally at a molecular level, which can be accomplished by regular monitoring of MRD. The aim is to detect leukemic cells in a background of normal hematopoiesis down to a level from 1:10^2^ to 1:10^6^ cells depending on the method applied. The evaluation of MRD is validated to identify patients at risk for relapse who are in morphological remission prior transplant [36]. In addition, it can also serve as a trigger for pre-emptive therapeutic interventions after transplant in order to prevent overt hematological relapse. In the following part we give an overview about methodical approaches for MRD detection consisting of molecular techniques, chimerism analyses and multi-parameter flow cytometry (Table 1).

## 4. Molecular MRD Assessment

The genetic heterogeneity of AML as previously described provides several disease-specific markers for MRD detection after allo-SCT. Molecular assessment of MRD can be performed by monitoring (1) mutated genes, (2) fusion gene transcripts and (3) overexpressed genes. PCR-based techniques to quantitatively measure these markers currently represent the standard of care with the highest sensitivity (down to 1:10^6^ cells) and specificity. This will be further improved in the near future by the introduction of sensitive next-generation sequencing (NGS) assays and digital-droplet based PCR as routine techniques for molecular MRD monitoring [15,37].

## 5. Detection of Gene Mutations for MRD Assessment

About 30% of patients with normal karyotype AML expose a mutation in *NPM1* (*Nucleophosmin 1*) gene, which is assumed to be relatively stable during disease course hereby fulfilling a major requirement for a suitable MRD marker [11,38,39]. Several groups already demonstrated the negative prognostic impact of increase in peripheral blood (PB)/bone marrow (BM) samples after conventional chemotherapy on relapse incidence and overall survival in NPM1-mutated AML, as recently reviewed [40,41,42,43,44]. Furthermore is was recently shown that MRD-positivity documented either by qRT-PCR or by NGS also has a negative prognostic impact on relapse-free and overall survival after allo-SCT [42,45,46,47]. Molecular testing for FLT3 mutations is part of the diagnostic setting at the time of first diagnosis and offers important prognostic information [1,48]. However, it is not routinely used to monitor MRD after allo-SCT due to its relative instability during disease course and a lack of a broadly available quantitative assay [49,50,51]. Generally, almost all mutations in the commonly mutated genes in AML may theoretically be measurable for MRD detection but have not been integrated into routine care yet. Indeed, due to their relatively low frequency being only present in small patient subgroups these markers lack a broad applicability and have therefore not been studied in larger cohorts. So far, only one report addressed the value of monitoring known DNMT3A and/or IDH1/2 mutations by digital droplet PCR in a small group of patients [49]. Representing an additional limitation, some of the above-mentioned so-called pre-leukemogenic mutations such as TET2, DNMT3A and ASXL1 may be retained at remission after chemotherapy and their prognostic impact is uncertain [52,53,54,55,56,57]. Furthermore, the investigation of AML-specific mutations by NGS after transplant may confront us with the phenomenon of donor-derived CHIP, a state with unknown impact on the risk for the development of donor-cell leukemia [58].

## 6. Detection of Fusion Gene Transcripts for MRD Assessment

In about 20% of patients with AML, excluding acute promyelocytic leukemia (APL), distinct fusion genes are approachable for MRD monitoring with most of them represented by core-binding factor (CBF) AML (RUNX1-RUNX1T1 and CBFB-MYH11 fusion genes) [1,12,13,59]. Several reports already demonstrated that MRD-positivity detected by quantitative PCR is predictive for imminent relapse in patients with CBF leukemias after conventional therapy [60,61,62,63]. Due to the low frequency of patients who receive allo-SCT in this good-risk AML category the evidence for MRD monitoring of CBF fusion genes in the post-transplant period is restricted to a small number of reports. As an example, Wang et al. reported a significantly higher cumulative incidence of relapse and shorter leukemia free survival for pts with MRD persistence after allo-SCT depicted by RUNX1-RUN1T1-positivity in PCR [64]. Besides CBF leukemias there are several other genetic rearrangements that are also accessible for post-transplant MRD monitoring such as t(9;11)(p21.3;q23.3)/MLLT3-KMT2A, t(6;9)(p22;q34)/DEK-NUP and inv(3)(q21q26)/RPN1-MECOM, but again their use is restricted to a low number of individuals with these molecular alterations [12,13,65].

## 7. Quantification of Gene Expression for MRD Assessment

In contrast to the limited frequency of individual gene mutations and fusion-genes mentioned above overexpression of Wilms Tumor 1 (*WT1*) mRNA is present in about 90% of patients with AML and 50% of patients with MDS. Thus, monitoring of WT1 is broadly applicable in a large proportion of AML and MDS patients [66,67]. Furthermore, *WT1* expression is measurable in peripheral blood with an even higher sensitivity and specificity than in bone marrow thereby facilitating high patient comfort in contrast to other methods for molecular MRD monitoring that require BM biopsy to gain a comparable sensitivity. As an additional advantage, *WT1* expression can be performed using a standardized, European LeukemiaNet (ELN) certified assay that offers a validated and reproducible cut-off level and comparability of results among different laboratories [66]. Several studies including one from our group recently demonstrated that longitudinal monitoring of PB *WT1* expression offers high sensitivity and specificity concerning detection of imminent relapse and appeared favorable compared to other methods for MRD monitoring such as cytogenetics, NGS-based molecular testing or chimerism analyses [68,69,70]. As a consequence, measurement of *WT1* is valuable option for MRD detection in patients with AML, at least in those cases where mutations or fusion genes are not accessible for sensitive PCR-based approaches [15,37].

## 8. Chimerism Analyses for MRD Assessment

Donor/recipient chimerism analysis is the standard practice to monitor donor cell engraftment and can be performed in all patients after allo-SCT. Analysis of chimerism also complementary augments MRD measurement and relapse prediction after transplant, even though it reflects not a direct proof of malignant cells by a leukemia-specific marker. Chimerism analysis detects host-derived hematopoiesis on the basis of genomic differences at highly variable gene loci between the recipient and the donor and this cannot directly be equated with relapse of the leukemic clone in all cases. However, in malignant disorders such as AML decrease of donor chimerism is often associated with disease recurrence [15,37]. Apart from this method-inherent limitation chimerism analysis has further restrictions. The conventional and the most widely adopted method using fragment analysis of short tandem repeats (STR) offers a sensitivity of 1 × 10^−2^ to 1 × 10^−3^ only [71,72,73]. This also applies for XY-FISH analysis in sex-mismatched donor/recipient constellations which provides a similar low sensitivity of only about 1 × 10^−2^ to 1 × 10^−3^ [74]. By employing variant-allele-specific quantitative PCR-based approaches to detect small DNA insertions or deletion sensitivity can be increased to a level with 1 × 10^−4^ to 1 × 10^−5^ cells [75,76]. Sensitivity and specificity of chimerism analysis can also be improved in patients with AML and MDS by evaluating the CD34+ cell subset [72,77]. Overall, chimerism analysis should be routinely performed after allo-SCT in conjunction with other more sensitive methods in order to identify patients at risk for relapse and to guide preventive interventions.

## 9. MRD Assessment by Multiparameter Flow Cytometry (MFC)

MFC is a standard MRD method to directly identify residual leukemic cells and can be performed in >90% with AML [37]. Two separate MFC approaches are capable to detect AML cells: the leukemia associated immunophenotype (LAIP) method defines a disease-specific expression pattern at diagnosis and facilitates subsequent tracking of this phenotype during follow-up period. If information about the immunophenotype at diagnosis is not available or if the occurrence of new or the disappearance of primary alterations are suspected, the different from normal (DfN) approach can be exerted [15]. These two approaches facilitate MRD assessment reaching a sensitivity of 10^−3^ to 10^−4^ [15]. To achieve optimal results, sensitivity and specificity an international expert panel recently recommended to use BM as primary material for examination, to use a minimum of 8 colors and to analyze the first BM pull to avoid hemodilution [15]. Several mostly retrospective reports have demonstrated the prognostic impact of MRD detected by MFC in patients with myeloid neoplasms after allo-SCT showing a significantly higher relapse risk for the patients with MRD-positivity compared to those without evidence for MRD by MFC [47,78,79,80,81]. Despite the main advantages of broad applicability and high sensitivity there still remain relevant limitations of this method in terms of a lack of comparability and reproducibility among different laboratories, the use of different instruments, fluorophores, and operating procedures that require further standardization [15,37].

## 10. Prevention and Treatment of Relapse after Allo-SCT

**Figure 2:**
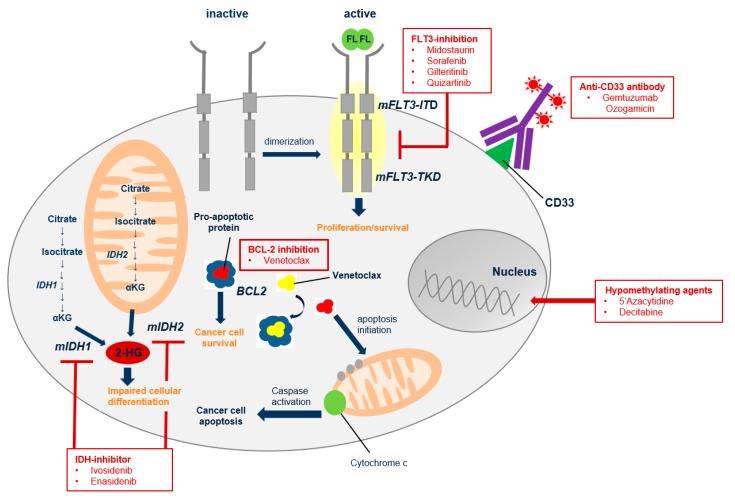
Potential targets for prophylactic and therapeutic interventions after allogeneic stem cell transplantation (allo-SCT) in patients with acute myeloid leukemia (AML). mFLT3-ITD (mutant FMS-like tyrosine kinase 3-internal tandem duplication); mFLT3-TKD (mutant FMS-like tyrosine kinase 3-tyrosine kinase domain); FL (FLT3 ligand); bcl2 (b-cell lymphoma 2); IDH1 (isocitrate dehydrogenase 1); IDH2 (isocitrate dehydrogenase 2); αKG (alpha ketoglutarate); mIDH1 (mutant isocitrate dehydrogenase 1); mIDH2 (mutant isocitrate dehydrogenase 2); 2HG (2-hydroxyglutarate)

Treatment of AML relapse after transplant is challenging due to a high rate of patients who either cannot tolerate intensive therapies as a consequence of toxicity of the previous transplant or do not achieve durable remissions by any treatment [3,6,8]. In general, any treatment for relapse of AML after transplant aims to deliver direct antileukemic activity and/or to enhance the immunological graft-versus-leukemia (GvL) effect. On the one hand, those therapeutic approaches consist of cellular-based therapies such as donor lymphocyte infusions [4] and second transplant in individual patients, which have been recently reviewed elsewhere [82,83]. On the other hand, pharmacological treatments represent the second backbone of relapse therapy and are often administered before any cellular therapy in order to reduce the leukemic burden. Traditionally, pharmacological approaches have mainly consisted of low-dose or intensive chemotherapy. However, as exemplified by a large registry-based analysis, the response to chemotherapy is limited (CR rate mild chemotherapy 17%, intensive chemotherapy 27%) and not all patients relapsing after transplantation can tolerate another round of intensive chemotherapy. Remissions after chemotherapy are generally not long-lasting if given alone. Those patients achieving CR after chemotherapy substantially benefit from donor cell-based consolidation. Indeed, 2-year OS was 55% in CR patients receiving donor lymphocyte infusion (DLI) or second transplantation compared to 20% in those without donor-cell treatment [8]. During the last years, hypomethylating agents, in particular Azacitidine (Aza) have proven to be a well-tolerated and efficient treatment alternative in this setting [35,84,85]. Furthermore, the decipherment of the molecular landscape in AML has facilitated the still ongoing development of targeted therapies. Some of them have already been licensed like the FLT3 inhibitor Midostaurin (EMA- and FDA-approved) and Ivosidenib and Enasidenib, inhibitors of IDH1 and IDH2, respectively [16,17,18], while others like the BCL2 inhibitor Venetoclax are still under investigation [19,20,21,86] (Figure 2). Based on the time point of intervention they can be divided into prophylactic approaches started when no evidence for leukemia is present, preemptive approaches which are initiated at the time of MRD detection to avoid frank hematological relapse and those approaches administered at the stage of hematological relapse.

## 11. FLT3 Inhibitors

Mutations in the gene encoding for the FMS-like tyrosine kinase 3 (FLT3) are present in about 30% of adults with newly-diagnosed AML. Almost three quarters of these patients have so called internal tandem duplications (ITD), which are mostly located in the juxtra-membrane domain of the receptor and result in a duplication of between 3 and more than 100 amino acids. The remaining 25% of patients have a point mutation in the tyrosine kinase domain (TKD) of the receptor [32,87]. While FLT3-ITD mutations, in particular those with a high mutant-to-wildtype allelic ratio (≥0.51), confer an adverse prognosis, the prognostic impact of TKD mutations is uncertain [14,32,87]. Both mutations lead to a constitutive, ligand-independent activation of the FLT3 receptor which mediates a continuous proliferative stimulus on leukemic cells. Giving this pivotal role for the pathogenesis of AML and the relatively high frequency of FLT3 mutations small-molecule FLT3 inhibitors have been developed. The first-generation of these molecules such as Midostaurin, Sorafenib, and Lestaurtinib were rather multi-kinase inhibitors than specific FLT3 inhibitors. This property to additionally inhibit several other kinases may explain the limited efficacy when administered as single agent and some of the off-target effects of these substances, which may either be undesirable in terms of side effects but also of interest with regard to potential efficacy in FLT3 wildtype AML [88,89]. In a large, placebo-controlled randomized phase-III trial Midostaurin was the first agent to show benefit with regard to overall survival which resulted in the approval of Midostaurin for first-line treatment (induction, consolidation and maintenance therapy) of patients with FLT3-mutated AML. In combination with conventional chemotherapy Midostaurin led to a 22% risk reduction for death and in particular those patients transplanted in first complete remission (CR1) seemed to benefit. Unfortunately, the role of Midostaurin as maintenance therapy after allo-SCT was not addressed in this trial and therefore Midostaurin is only licensed as maintenance therapy after conventional chemotherapy [18]. Meanwhile, second generation molecules such as Gilteritinib and Quizartinib, which inhibit FLT3 more specifically, have been developed and appear to be more effective even when used as single agents [88,90]. Updated results from the latter randomized phase-III trial in relapsed/refractory AML patients with FLT3-ITD mutation have recently been updated and show prolonged overall survival 0.76 (95% CI, 0.58–0.98; *p* = 0.0177) and a higher composite complete remission (CR) rate (48% vs. 27%) for Quizartinib in comparison to salvage chemotherapy. Both of these trials included patients who were treated for relapsed AML after allo-SCT but did not report the details of these subgroups separately. Moreover, gilterinib is currently tested as maintenance therapy after allo-SCT in an ongoing, randomized, placebo-controlled phase III trial, where patients are envisaged to receive maintenance therapy with 120 mg Gilteritinib per day for 24 months (EudraCT #2016-001061-83). Patients are planned to start maintenance therapy early after allo-SCT between day 30 and day 90, but as a particular strength they are already registered prior transplant. The latter enables estimation of drop-out rate prior to the start of maintenance therapy and thereby the interpretation how many patients will finally be able to start and tolerate maintenance with an FLT3 inhibitor after allo-SCT. Furthermore, Crenolanib is another highly selective and potent FLT3 tyrosine kinase inhibitor (TKI) with activity against ITD mutants and FLT3/D835 point mutants [91]. Crenolanib is currently under investigation as salvage therapy in relapsed/refractory patients [92]. Still, a randomized phase-III trial comparing Crenolanib with standard salvage therapies (NCT02298166) has been stopped this year due to strategical considerations of the company. Despite this, investigation of Crenolanib as maintenance therapy after allo-SCT (NCT02400255), but also in addition to first-line chemotherapy in a head-to-head comparison to midostaurin (NCT03258931) is ongoing. Another interesting option regarding the use of crenolanib in AML patients may be the combination with CAR T-cells, since preclinical analyses revealed a synergistical effect of FLT3 targeting CAR T-cells and crenolanib [93]. A synergistical effect of crenolanib has also been demonstrated for the combination with azacitidine. In this context, azacitidine renders FLT3+ AML cells more sensitive to crenolanib by effectively abrogating stromal protection [94]. As salvage therapy for relapsed FLT3-mutated AML most evidence comes from the use of Sorafenib. The knowledge about Sorafenib is again based on retrospective case series and reports, but not controlled prospective trials [95,96,97]. The use of Sorafenib in FLT3-mutated AML after allo-SCT was performed as off-label use on an individual basis facilitated by the availability of Sorafenib as licensed drug for the treatment of renal and liver cancer. Nevertheless, in these reports monotherapy with Sorafenib seems to induce durable remissions and long-term survival in a subgroup of patients and the anti-leukemic effect of Sorafenib seems to synergize with allo-immune effects. As a mechanistic explanation, Mathew et al. recently discovered that Sorafenib increases IL-15 production by FLT3-ITD+ leukemia cells in mice and human thereby inducing leukemia-reactive T cells [98]. To take advantage of these properties Sorafenib has also been tested as maintenance therapy after allo-SCT in a randomized phase II trial (EUDRACT 2010-018539-16). Data from this well-designed, randomized trial have recently presented at the annual meeting of the American Society of Hematology and suggest a relevant benefit of Sorafenib maintenance therapy in terms of overall and relapse-free survival [99]. Even though not licensed in this indication, this may have practical implications when considering maintenance therapy in FLT3-mutated patients.

## 12. Isocitrate Dehydrogenase (IDH) Inhibitors

Another interesting molecular target also in the context of allo-SCT are mutations of the cytosolic IDH1 (R132) and the mitochondrial IDH2 (R140 and R172) enzyme which can each be found in about 5–15% of patients with AML. Usually, these enzymes catalyze the transformation from isocitrate to α-ketoglutarate. Mutations in these enzymes found in patients with AML result in a neomorphic enzyme activity which now produce 2-hydroxyglutarate. This oncometabolite impairs cellular differentiation in hematopoietic stem and progenitor cells and thereby substantially contributes to the pathogenesis of AML [16,100,101]. Taking this into account IDH inhibitors have been developed in order to target these mutations selectively. The orally available inhibitors (IDH1, ivosidenib; IDH2, enasidenib) have initially been tested in patients with relapsed/refractory patients with AML and demonstrated encouraging rates of complete remissions (CR, CR + CR incomplete: ivosidenib 30.4%; enasidenib 26.1%) [16,17]. Based upon these results, both drugs were approved by the FDA for the treatment of relapsed/refractory AML and are now tested in the front-line setting either in combination with intensive chemotherapy or with hypomethylating agents. Their unique mode of action conferring antileukemic activity by the promotion of differentiation instead of a primary cytotoxic effect resulted in a limited and class-specific toxicity profile [16,17,101,102].

Both, the acceptable toxicity and the mode of action imply that these substances may also be an interesting option for patients with IDH-mutated AML who have relapsed after allo-SCT. In detail, by inducing cellular differentiation the IDH inhibitors may promote an allo-immunologic reaction by antigen upregulation on leukemic cells thereby enhancing their immunogenicity. Although both trials in patients with relapsed/refractory AML also included a limited number of patients with relapse after allo-SCT, their specific outcome has not been reported in detail so far.

To explore the role of IDH inhibition either as maintenance and or as salvage therapy also after allo-SCT more in detail, there are now several prospective studies including one from our group on the way.

## 13. Hypomethylating Agents and HDAC Inhibitors

Unfortunately, apart from those with IDH and FLT3 mutations many AML patients have no molecular alteration that can currently be therapeutically addressed by a targeted approach prior to transplant as well as to prevent or treat relapse after transplant. In addition, even if present before transplant, a specific molecular target, such as FLT3 mutations, may have been lost after transplant as a process of clonal evolution [103,104]. Conversely, new molecular lesions may occur at relapse, leading to the need to perform extensive molecular examinations in cases of AML recurrence. Thus, a preventive or therapeutic approach after transplant may rather be a more general option for a relevant proportion of patients if it is effective independent from any molecular structure or biological features.

The hypomethylating agents Azacitidine (Aza) and Decitabine (DAC) seem to fulfill many of these criteria and have intensively been investigated during the last years. They mediate a direct anti-leukemic effect in patients with AML and MDS independent from a distinct molecular phenotype, seem to beneficially influence the balance between GvL effect and GvHD and are not associated with an excess of toxicity [90,105,106,107,108]. In detail, by upregulating epigenetically silenced leukemia antigens such as cancer-testis antigens [109] and the re-expression of endogenous retroviral elements and dsRNA causing an antiviral interferon response they increase the immunological visibility of AML and MDS cells [110]. Furthemore, HMA seem to favorably modulate the activity of T- and NK cells towards an enhanced GvL effect, which is not counterbalanced by an increased risk of GvHD [111,112,113].

To take advantage of these properties the HMA Aza (*n* = 4) or DAC (*n* = 2) have been tested as maintenance therapy to avoid relapse after allo-SCT in 5 prospective single-arm studies and one retrospective case series covering a total of 148 patients [111,114,115,116,117,118]. Those early-phase trial generally demonstrated feasibility and identified the optimal dosage and schedule for future trials, which was lower than the one recommended for the primary indication was generally administered in these trials. Indeed, Aza (32 mg/m^2^ per day, day 1 to day 5) is currently tested for relapse prevention after allo-SCT in an ongoing, randomized placebo-controlled trial in patients with high-risk AML or MDS (NCT00887068). Results from this trial are awaited next year.

Another interesting option in this context is the use of an oral formulation of Aza (CC-486). This compound is currently tested in a large company-driven randomized, placebo-controlled trial as maintenance therapy in elderly AML after induction chemotherapy (NCT01757535) and has also been recently tested as maintenance therapy after allo-SCT in a single-arm phase I/II dose finding study including 31 patients with AML or MDS [119]. Advantages of this formulation may be a better patient compliance and convenience, due to outpatient administration as well as its pharmacokinetic and pharmacodynamic properties facilitating prolonged exposure and sustained DNA hypomethylation.

Aza and DAC have also been tested in several retrospective analyses and in 4 small-sized prospective studies as salvage therapy either alone or in combination with DLI in patients with relapse of AML or MDS after allo-SCT. As recently reviewed elsewhere [85,120,121], results from these analyses clearly demonstrated that this well-tolerated combination of a HMA and donor cells can induce durable remissions (CR rates ranging from 10% to 75%) and long-term survival in a relevant proportion of patients (2-year survival rates ranging from 12% to 80%). In particular patients with a diagnosis of MDS and those treated at the stage of molecular relapse instead of hematological relapse seems to benefit from this combination highlighting the need for stringent MRD monitoring [35,122]. This concept of pre-emptive therapy at the time of molecular relapse after allo-SCT has been tested by Platzbecker and colleagues in two prospective trials. In an initial proof-of concept study (RELAZA-1) 20 patients with MDS or AML were treated with up to 4 cycles Aza as soon as the CD34+ donor chimerism dropped in peripheral blood below a threshold of 80%, while patients were still in hematological remission. Despite an improvement of chimerism (>80%) in half of the patients, this early intervention was able to induce durable remissions only in 3 (30%) of the responders and did not avoid progression towards hematological relapse in the majority of patients [122]. They expanded this analysis in a second trial (RELAZA-2) covering 53 patients (24 after allo-SCT, 29 after conventional chemotherapy), who were monitored by CD34+ donor chimerism or molecular markers such as NPM1 and RUNX1-RUNX1T1. In case of MRD positivity patients could receive up to 24 cycles Aza. The study met its primary endpoint with 31 patients (58%) free of relapse after 6 months and 19 of 53 patients achieved a major response (36%). However, similar to their first trial treatment with Aza, even though with an expanded number of cycles, could only delay relapse in many patients and 26 patients (49%) finally relapsed [123]. In those patients after allo-SCT this is probably related to the fact that DLI were not part of the protocols of both trials. Although especially the combination of Aza and DLI has been established as valuable treatment alternative besides chemotherapy and second transplantation, the responses rates in particular in patients treated at the stage of hematological relapse indicate there is still space for improvement [35]. Here, the combination of Aza with a targeted approach may be an option to improve outcome in patients with relapse after allo-SCT. In this context, DiNardo and colleagues recently reported encouraging response and survival data in treatment-naïve elderly AML patients treated with HMA in combination with Venetoclax, a potent, selective oral inhibitor of the antiapoptotic b-cell lymphoma 2 (BCL-2) protein. Response rates and overall survival seemed to be higher than observed after monotherapy with HMA making this combination an attractive option also for patients, including subjects post allo-SCT [19]. This also applies to the combination of HMA and FLT3 inhibitors such as Sorafenib, which has also been demonstrated to induce long-term remissions in individual patients [124,125,126].

Besides HMA also the HDAC inhibitor Panobinostat has demonstrated feasibility as maintenance therapy after allo-SCT in 42 patients with AML or high-risk MDS in an open-label, multi-center phase I/II trial. Consequently, Panobinostat is currently investigated in a large randomized trial.

## 14. Cellular Therapies

### 14.1. Donor Lymphocyte Infusions

Besides pharmacological anti-leukemic approaches (e.g., chemotherapy and targeted therapies) cellular interventions represent the second backbone to prevent and to treat relapse of AML after allo-SCT. Donor lymphocyte infusions (DLI) are a cellular product of mononuclear cells containing a defined number of donor-derived CD3+ T cells. DLI can be obtained either as aliquots from the initial G-CSF-mobilized PB stem cell product or by an unstimulated leukapheresis of the original donor [127]. Following an initial description in 3 patients with chronic myeloid leukemia [128] the application of DLI has also been extensively employed in patients with AML [127]. Likewise in CML the aim is to enhance the GvL effect in order to either prevent relapse in case of remission after transplant (e.g., prophylactic DLI) or in case of disease recurrence to treat relapse (e.g., therapeutic DLI). Several retrospective analyses and a limited number of prospective studies have reported on the use of DLI as prophylactic approach. In T-cell depleted (TCD) setting the beneficial effect of prophylactic DLI on progression-free and overall survival is well established [129,130,131]. In the non-TCD setting prophylactic DLI also seemed to improve the outcome of high-risk AML patients after sequential conditioning using the FLAMSA-RIC regimen when compared to historical controls [132,133]. Prophylactic DLI were given to 46 patients with high-risk AML starting from day +120 after allo-SCT if patients were in still in remission, off immunosuppression for 30 days and free of GvHD. After a median follow-up of 7.2 years OS was 67% at 7 years in those patients receiving prophylactic DLI compared to 31% in the control group [133]. These data have been supported by a recent matched-pair analysis from the EBMT registry [134]. Nevertheless, the retrospective character of most analyses and the relatively late timepoint of DLI application suggest a relevant selection bias. To overcome the potentially higher GvHD risk associated with earlier application Wang et al. administered G-CSF mobilized DLI by day +40–60 followed by short-term GvHD prophylaxis [135]. The lack of randomized prospective trials and the heterogeneity of the retrospective data in different settings do not facilitate a clear recommendation on indication, timing, and dosage of prophylactic DLI. Data regarding therapeutic DLI are also mainly based on retrospective analyses but also suggest a limited efficacy, as indicated by a 2-year OS rate of 21% in relapsed patients receiving DLI compared 9% in those not receiving DLI [7,136]. The success of therapeutic DLI strongly correlated with disease burden at relapse and remission state at the time of DLI application [7,136]. This implies the need for regular MRD monitoring for early relapse detection but also highlights the need for efficient salvage therapies to induce remission prior to DLI.

### 14.2. Second Transplantation

Apart from DLI, another form of cellular therapy is a second allogeneic transplantation after anew conditioning followed by immunosuppression. In a large retrospective analysis of 179 patients with acute leukemia (AML *n* = 132, acute lymphoblastic leukemia *n* = 46) the 2-year OS rate after second allo-SCT was 25% in the entire cohort [5]. Second transplantation in CR, an interval of >6 months between first allo-SCT and relapse and a matched related donor at first transplantation were associated with a better outcome in multivariate analysis. Assuming that the GvL effect mediated by the first donor was insufficient to control leukemia a donor change at second transplant is worth considering, although in the analysis by Christopeit et al. no significant impact of donor change could be demonstrated in multivariate analysis. Given the advances in the field of haploidentical transplantation a second allo-HSCT using a haploidentical donor may also be an option that is currently employed in individual patients [137]. However, in this context a comprehensive analysis of the HLA constellation of the recipient and donors is required to guide donor selection, as loss of HLA may have driven relapse after allo-SCT 1 [138,139,140]. Finally, it needs to be taken into account that patients included in these retrospective analyses represent a positively selected cohort that survived until intervention. Furthermore, as exemplified by a median age of 38.5 years at allo-SCT2 in the report of Christopeit et al. [5], patients receiving a second transplant are mostly younger than the majority of AML patients relapsing after allo-SCT1 and thereby fit enough to tolerate such an intensive therapy. Based on this, the decision on a second transplantation can only be made individually by carefully weighting the risks and chances of this procedure.

### 14.3. New Cellular Therapies

Alloreactivity of DLI and second transplantation is mainly mediated by recognition of disparities in minor histocompatibility antigens in the matched donor setting and of HLA disparities in case of mismatched donors. Current investigations aim to improve the efficacy and toxicity profile of cellular therapies by selection of specific immune cells (e.g., NK cells, γ/δ T cells etc.) or by manipulation of the cellular product (e.g., external cytokine stimulation or genetic modification) [127]. In this context, chimeric-antigen-receptor (CAR)-modified T cells are also under investigation for patients with AML and initial data seem promising [93]. However, in contrast to patients with B cell malignancies the transfer of CAR T cells from bench to bedside in patients with AML is slower. This is mainly related to the difficulty to identify AML-specific target epitopes, which are expressed as few as possible on normal tissues, but homogenous on AML cells of a larger proportion of patients and exert an essential function for AML biology. Among others, current targets of interests consist of CD123, CD33, folate receptor β, FLT3, CLL-1 and CD44v6 [141].

## 15. Conclusions

During the last decade, our understanding of the molecular heterogeneity and pathophysiology of AML has been greatly advanced by the use of high-throughput sequencing techniques. The discovery of several recurrently mutated genes and chromosomal aberrations has markedly improved the classification and risk stratification. Furthermore, first steps towards a more targeted therapy for patients with AML have been made by the introduction of Midostaurin and several other compounds will probably follow in the near future. This knowledge about molecular alterations and the opportunity to monitor MRD has, similar to the pre-transplant phase, already been adopted into routine care after transplant. While randomized studies investigating maintenance strategies with HMA or targeted approaches such as FLT3 inhibitors are currently ongoing, we are already using such compounds to treat patients with relapse after transplant. Until those studies will have demonstrated a benefit of maintenance therapy, we suggest to employ the molecular information in individuals patients for stringent MRD monitoring and to perform preemptive therapeutic interventions guided by disease phenotype and genetics.

## Figures and Tables

**Table 1:** Methods to detect Minimal Residual Disease (MRD) in Patients with AML after allo-SCT.

	Multiparametric Flow Cytometry	Molecular Genetics (Fusion Transcripts, Point Mutations, Gene Overexpression)	Chimerism
Methods/Approaches	LAIP/DfN	qPCR // digital droplet PCR (ddPCR) // NGS	qPCR // Indel-PCR // STR-based // XY-FISH
Sensitivity	10^−3^–10^−4^	10^−3^–10^−6^	10^−2^–10^−3^ 10^−4^ –10^−5^
Advantages	broad applicability	high sensitivity and specificity	applicable in all patients after allo-SCT
Disadvantages/Perspectives	need for standardization	mostly restricted to a small part of patients need for standardization MRD monitoring based on mutations not yet established	low sensitivity and specificity not directly detecting leukemic cells CD34+ sorted chimerism offers increased sensitivity

DfN (different from normal); ELN (European LeukemiaNet); FISH (fluoreszenz in-situ hybridization); Indel (insertion and deletions); LAIP (Leukemia-associated immunephenotype); NGS (next generation sequencing); pB (peripheral blood); qPCR (quantitative polymerase chain reaction); STR /short-tandem-repeats).
